# Statistical explanation of the protective effect of four COVID-19 vaccine doses in the general population

**DOI:** 10.3389/fpubh.2023.1253762

**Published:** 2023-09-22

**Authors:** Humberto Reyes, Constanza Méndez, Alexis M. Kalergis

**Affiliations:** ^1^Millennium Institute on Immunology and Immunotherapy, Santiago, Chile; ^2^Departamento de Genética Molecular y Microbiología, Facultad de Ciencias Biológicas, Pontificia Universidad Católica de Chile, Santiago, Chile; ^3^Departamento de Endocrinología, Facultad de Medicina, Escuela de Medicina, Pontificia Universidad Católica de Chile, Santiago, Chile

**Keywords:** COVID-19, vaccination, ICU hospitalizations, explanatory model, GAMLSS

## Abstract

**Objectives:**

To assess the effectiveness of four doses of the vaccine against SARS-CoV-2 in the general population and the impact of this on the severity of the disease by age group.

**Methods:**

By using data from the health authority public data base, we build statistical models using R and the GAMLSS library to explain the behavior of new SARS-CoV-2 infections, active COVID-19 cases, ICU bed requirement total and by age group, and deaths at the national level.

**Results:**

The four doses of vaccine and at least the interaction between the first and second doses were important explanatory factors for the protective effect against COVID-19. The *R*^2^ for new cases per day was 0.5644 and for occupied ICU beds the *R*^2^ is 0.9487. For occupied ICU beds for >70 years *R*^2^ is 0.9195 and with the interaction between 4 doses as the main factor.

**Conclusions:**

Although the increase in the number of vaccine doses did not adequately explain the decrease in the number of COVID-19 cases, it explained the decrease in ICU admissions and deaths nationwide and by age group.

## Introduction

The pandemic caused by SARS-CoV-2 between 2020 and 2022 has caused a major public health burden with large number of deaths worldwide (1). As of May 23, 2023, a total of 676,609,955 cases and 6,881,955 deaths have been recorded (2). Globally, an estimated 68.4% of the population has received at least one dose of one of the available COVID-19 vaccine (1). With vaccination, face mask usage and quarantines, contagion rates were reduced. However, new SARS-CoV-2 variants of concern (VOC) have emerged further increasing virus spread and need of a global health emergency declaration by the World Health Organization (WHO) (3). The Omicron variant (B.1.1.529) has caused worldwide concern due to its high transmissibility and the reduced protection generated by vaccines against infection with this variant (4). Fortunately, the beneficial effect of herd immunity produced by mass vaccination has led to a reduction of severe COVID19 cases worldwide allowing WHO to declare the end of sanitary emergency on May 2023 (5).

Longitudinal studies of efficacy and effectiveness for various vaccines have reported a decrease in neutralizing antibodies during follow-up, which generated the need to introduce booster doses in the population (6–8). In Chile, Phase 3 studies in adults immunized with two doses of an inactivated SARS-CoV-2 vaccine (CoronaVac^®^) separated by 14 or 28 days showed that immunization with this vaccine induced robust humoral and cellular immunity and that the 28-day schedule induced a stronger humoral immune response than did the 14-day schedule (9–12). Further, a fourth dose of a homologous scheme with CoronaVac^®^ managed to reestablish the neutralizing antibodies and maintain the cellular response against the wild type (WT) strain and Delta and Omicron (B.1.1.529) variants of SARS-CoV-2 (13). However, recent evidence is consistent in showing that the immune response triggered by original vaccines is lower for the Omicron variant and its subvariants as compared to the Wuhan SARS-CoV-2 strain (14). Therefore, booster vaccination campaigns for COVID-19 continue to be a priority for global public health (15, 16). When evaluating the effectiveness of vaccination against intensive care unit (ICU) admission, two-dose vaccination is less effective than three-dose vaccination, and the effectiveness drops from 68 to 36% if more than 2 months have passed since the last vaccination (17).

Chile has been one of the countries with the highest rates of vaccination against SARS-CoV-2 and, currently, 79.9% of this population has received a second booster (18). Previously, we built a model that explained the behavior of the pandemic data as a function of the vaccination, which at that time consisted of only two doses, and gave a central role to the number of doses administered and the interaction between these two doses (19). The hypothesis of this work is that the total number of doses of SARS-CoV-2 vaccine administered to the Chilean population contributed to the control of the pandemic. This was evaluated on the basis of the models developed in the previous work, with the objective of analyzing how the data behaved as the number of doses in the total population of Chile increased.

## Materials and methods

Public data provided by the Ministry of Health of Chile and the Ministry of Science and Technology of the same country were analyzed for this study (18). As an initial analysis, models were used to examine the evolution of key pandemic-related variables; (1) daily number of new COVID-19 cases; (2) daily active COVID-19 cases; (3) daily ICU bed occupancy; and (4) daily COVID-19-related deaths. The different models generated for each response variable were constructed based on successive combinations of the doses administered with the vaccines, including their interactions or the total amount of vaccines administered. That is, for each variable studied, a model was developed that considered the total number of vaccines administered without discrimination by dose. Other models were also generated for each of the following situations: with only the first dose, with the first and second doses, with the first, second and third doses, and with the four doses administered to the population. In addition, the various interactions that could occur between the different doses administered were included. In the analysis of each variable studied, models were generated with the different combinations of doses described above. This was done for the variable new cases per day. However, in the case of active cases, in addition to considering the different models with the doses administered, new cases per day was introduced as an additional factor. In the context of ICU bed occupancy, both new and active cases were added as factors. Furthermore, in the models explaining deaths, additional variants were generated that included new cases, active cases, and ICU bed occupancy as influential factors in their dynamics. These analyzes were performed considering the total population.

A second considered the population according to different age groups for the variables in which this information was available. The models were made using different combinations of de variables age groups (3–39 yo, 40–49 yo, 50–59 yo, 60–69 yo, and 70 and over yo), the total cumulative number of vaccines administered, the cumulative daily number of vaccines administered and of first, second, third, and fourth dose of the vaccine. All these factors were adjusted to weekly counts because of the periodicity with which the data were uploaded to the public database. All outcome variables were normalized to counts per 100,000 population. This comprehensive approach made possible to address the relationship between vaccination and key epidemiologic variables, taking into account both the doses administered and other factors influencing the dynamics of the pandemic, but did not take into account other factors such as hospitalization measures, other health measures, or comorbidities of individuals in intensive care or deceased, as these types of data were not available in the source from which they were obtained.

All generated models and their respective analyses are available in the Github repository https://github.com/Aujeszky/vaccination_with_4_doses.

For our outcome variables of interest, we used generalized additive models for location, scale, and shape (GAMLSS) using a Gamma distribution (which is appropriate for continuous variables, as is the case for the normalized outcome variables used here). The processing and analysis of the national dataset was automated in scripts written in the R programming language (20) and the models generated were analyzed using the GAMLSS library (21). Previously published criteria were used to select the best model (19). To analyze the evolution of the models and their influence on the different variables have influenced them, the best model for each case was taken and compared with its similar models, iterating over each day to obtain the Akaike Information Criterion (AIC) and *R*^2^ of each model. The *R*^2^-value was maintained, but the AIC values were normalized to the best model to facilitate comparison.

## Results

### Four vaccine doses reduce infection severity

In the Chilean population, 91.92% have received at least one dose of the SARS-CoV-2 vaccine, 87.03% have received at least two doses, 79.9% have received three doses and 59.72% have received four doses (Supplementary Figure 1A). The vaccine formulations administered in the Chilean population were those produced by Sinovac, Pfizer, Moderna, CanSino, and Astra-Zeneca. Out of these vaccines, Sinovac was the most massively administered vaccine for the first and second doses, and Pfizer vaccines were the most frequently administered for the third and fourth doses (Supplementary Figure 1B). To correctly analyze the results derived from the models, it must be considered that the national mass vaccination campaign started on February 3, 2021 and the administration of the second dose started 28 days later, in March 2021, with only 0.019% of the population vaccinated with the first dose; this group corresponds mainly to older adults. On August 11th of the same year, immunity was reinforced with a third vaccine dose, by which time 72.24 and 63.04% of the population had been vaccinated with the original first and second doses, respectively (22). One year after the start of the national vaccination campaign, in February 2022, the second booster (fourth vaccine dose) was administered to the population, at which time 89.8% of the population had been vaccinated with the first dose, 83.65% with the second dose and 64.1% with the first booster (or third vaccine dose) (Supplementary Figures 1A, B). When observing the number of cases per day, a peak was observed in January 2022, which coincided with the start of the second booster dose (Supplementary Figure 2A). On the contrary, the peak did not coincide with the number of occupied ICU beds, observing a slight increase in December 2022 that did not exceed the previous increases (Supplementary Figure 2C). Deaths associated with COVID-19 infection had dropped since October 2021, however, an increase was observed in February 2022 (Supplementary Figure 2E).

### Booster immunization led the decline in ICU bed occupation and deaths

**Figure 1:**
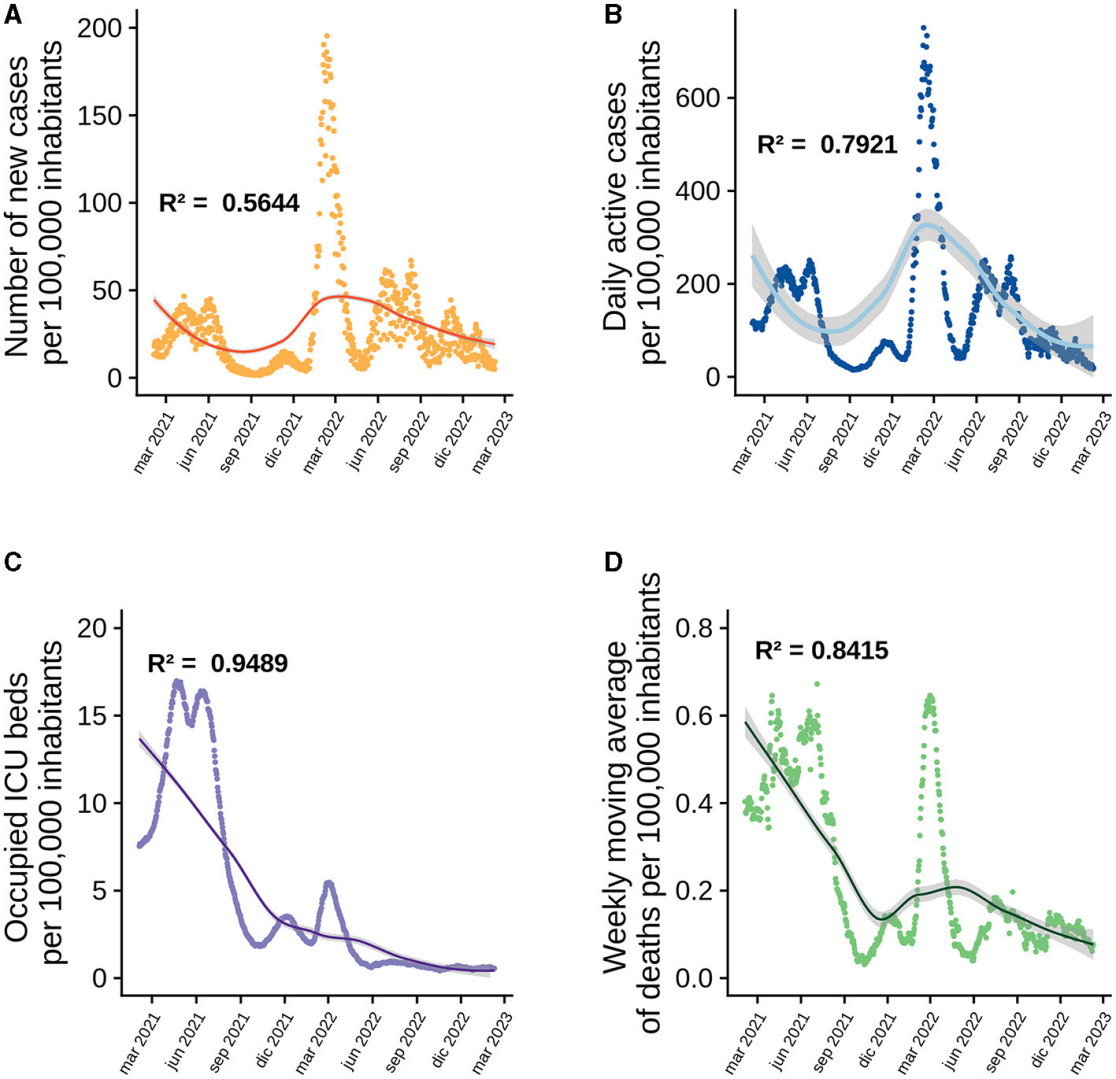
Behavior of national level data analyzed since the beginning of the national vaccination campaign using GAMLSS. In each graph the points correspond to daily count data per 100,000 population, the curves represent the model fit and the shaded area is the standard error of the model, each graph shows its corresponding *R*^2^. **(A)** New cases increase in 100,000 inhabitants over time. **(B)** Active cases. **(C)** ICU beds occupied by patients with COVID-19. **(D)** Deaths due to COVID-19.

**Figure 2:**
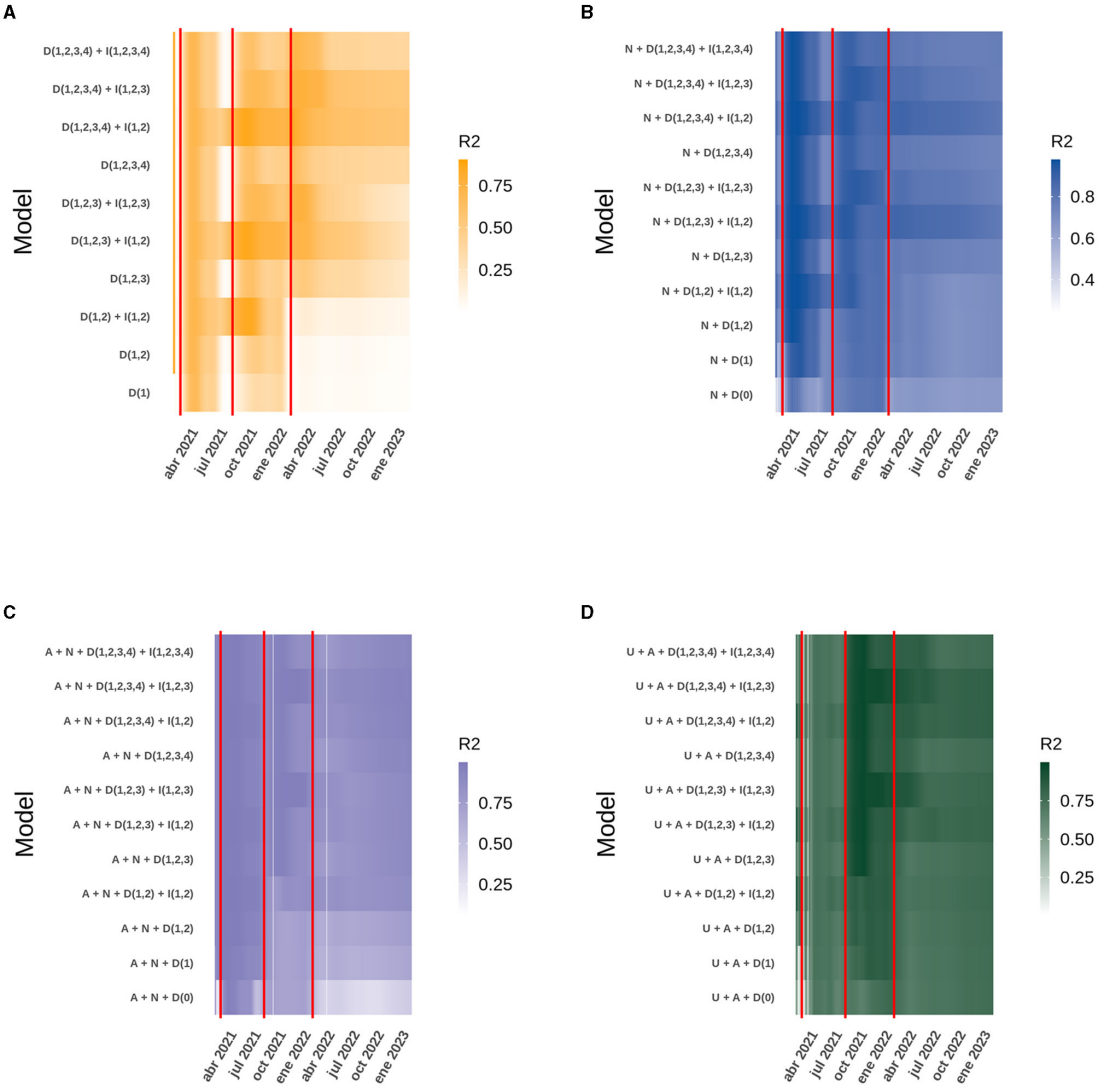
Heatmap of the daily *R*^2^ obtained from the best model compared to similar models for the data analyzed at the national level. The red vertical lines correspond to the dates on which the second, third and fourth doses of the vaccine were started. The letter code corresponds to: U (Daily ICU beds), A (Daily active cases), N (Daily new cases), D (Number of doses), I (Interaction between doses). **(A)** New cases. **(B)** Active cases. **(C)** ICU beds occupied by patients with COVID-19. **(D)** Deaths due to COVID-19.

To understand the impact of vaccination on the Chilean population throughout the pandemic, explanatory models were generated based on GAMLSS, evaluating the number of new COVID-19 cases, active cases, occupied ICU bed number and deaths, based on models generated with the same data provided by (18). As described previously (19), the best model at national level for each response variable always includes the doses administered and the interaction between the original doses. However, since a larger number of doses we given, new models are required to analyze the effect of all four doses and the statistical interaction that may occur between all four doses. Therefore, the variable “total vaccines administered” was also included in the models, which consists of evaluating the vaccination as a whole and not by dose, and no model that adequately explained the behavior of the data included this variable. The best model to explain the behavior of new cases from the start of the national vaccination campaign until February 8th, 2023, is the one that includes the four doses administered to the population over 3 years of age and the statistical interaction between the first and second dose. This model has an *R*^2^ = 0.5644 (Figure 1A), which decreases to 0.3866 when the interaction between the first and second dose is removed as an explanatory factor. The most important variables in this model are the third doses, followed by the interaction between first and second dose (Figure 2A). Active cases are explained by new cases, the four doses given and the interaction between the first two doses, although the model that includes new cases is very close to this model. The *R*^2^ of this model is 0.7921 (Figure 1B) and drops to 0.7305 when the interaction factor is removed. In addition, the performance of the model drops significantly when new cases are excluded, resulting in an *R*^2^ = 0.3224. The most important factors in the explanation of the behavior of active cases are, in order of importance: new cases and the third dose (Figure 2B). For the occupied ICU beds variable, the best model explaining the behavior of the data is the one that includes active cases, new cases, the four doses of vaccine and the interaction between the first three doses. This model has an *R*^2^ = 0.9489 and shows a downward trend from the beginning (Figure 1C). The most important factors in this model are the fourth dose and the interaction between doses one, two, and three (Figure 2C). To explain the number of deaths due to COVID-19 in Chile, active cases, ICU beds occupancy, the four vaccine doses and the interaction between the first three vaccines are the factors that best explain the behavior of the data, giving an *R*^2^ = 0.8415 (Figure 1D). Within this model, the most important factors are the interaction between first three doses and active COVID-19 cases (Figures 2A–D).

### Interaction between doses explains reduced ICU bed occupancy based on age range

**Figure 3:**
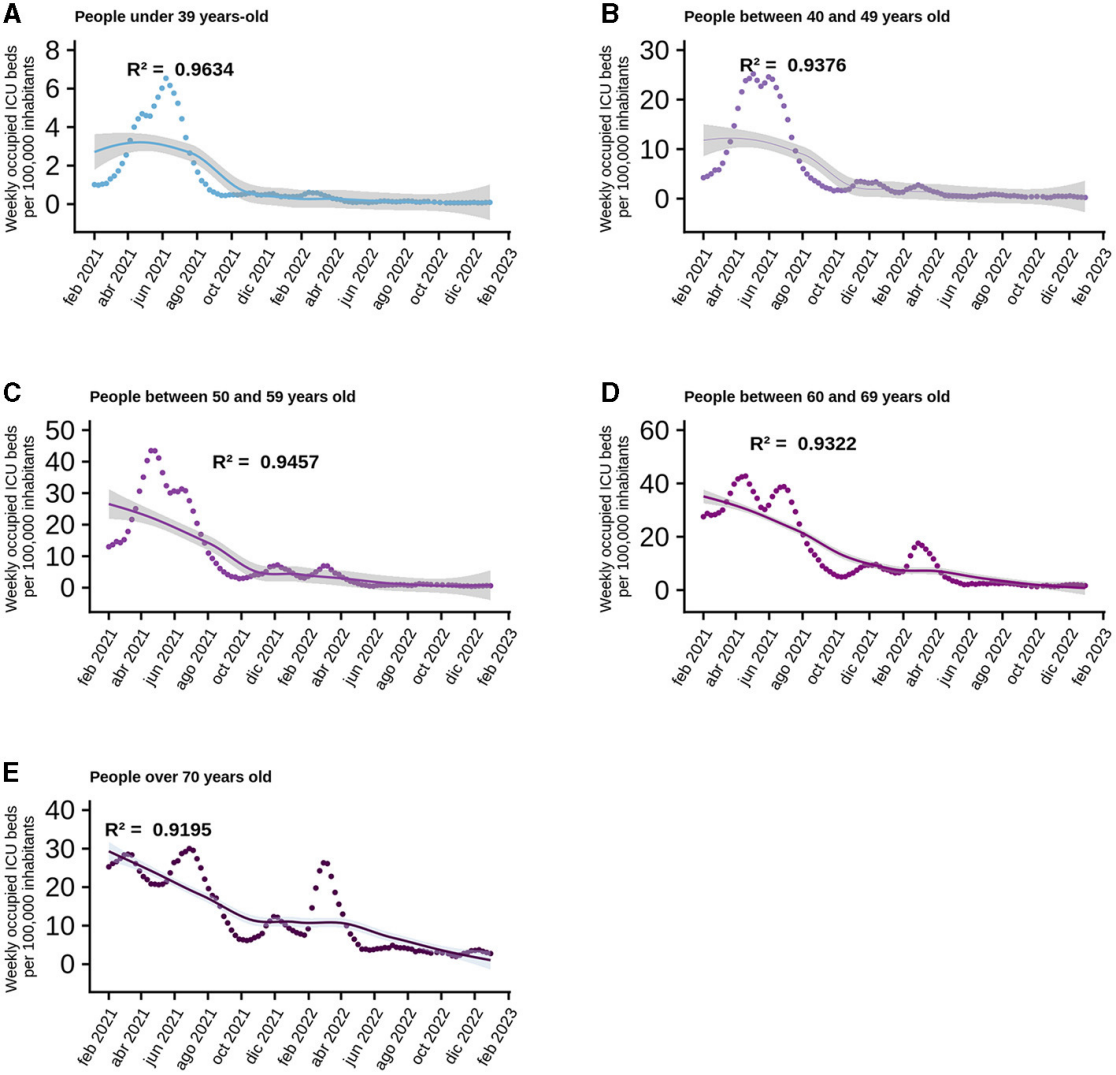
Behavior of normalized data per 100 thousand inhabitants of ICU beds according to age range. Analyzed since the beginning of the national vaccination campaign, the points correspond to the data provided by MINSAL on a weekly basis and the curve corresponds to the best model generated and the shaded area is the standard error of the model. Each graph shows the *R*^2^ for each of them: **(A)** Persons under 39 years-old. **(B)** People between 40 and 49 years old. **(C)** People between 50 and 59 years old. **(D)** People between 60 and 69 years old. **(E)** People over 70 years old.

At a national level, the model explaining the behavior of ICU bed occupancy due to COVID-19 has the best fit, so we wanted to use GAMLSS models to break down how the data behave according to the age range of ICU patients. The data used to generate the models only included new cases per week, total vaccines and vaccines administered to each age group, as daily data were not available, so the data were analyzed on a weekly basis. In the age group corresponding to persons under 39 years, the most parsimonious model includes as factors the new cases within the same group, the four doses of vaccine and the interaction between the first and second dose. The *R*^2^ of this model was equal to 0.9634 (Figure 3A) and has as the most important factor the interaction between the doses, followed by the new cases (Supplementary Figure 3A). For those aged 40–49, as for those under 39, the best model includes new cases, the four vaccine doses and the interaction between the first and second doses. This model has an *R*^2^ = 0.9376 (Figure 3B), which drops to 0.922 when the interaction is removed from the explanatory factors, but the drop is radical when only new cases are considered, resulting in a an *R*^2^ = 0.001 (Supplementary Figure 3B). The trend of the previous models was maintained in the 50–59 age group, with new cases, the four vaccine doses and the interaction between the first two doses. This model also explains the variability of the data very well, with an *R*^2^ = 0.9457 (Figure 3C), which decreased to 0.0001 when only new cases were considered as an explanatory factor (Supplementary Figure 3C). The scenario begins to change for people aged between 60 and 69, as the new cases and the four doses are still present in the best models, but now the interaction between the four doses explains the behavior of the data better than the interaction between the first two doses, giving an *R*^2^ = 0.9322 (Figure 3D). When the interaction was removed from the model, the *R*^2^ drops to 0.837, indicating the importance of the interaction at this level between the different vaccine doses (Supplementary Figure 3D). Something similar to the case for people aged 60–69 was observed for people aged 70 and more: the best model included the new cases, the four vaccine doses and the interaction between them, but the *R*^2^ was only 0.9195 (Figure 3E), the most important factor in the model was the interaction between the doses (Supplementary Figure 3D).

### The interaction between the doses explains the behavior of all variables over time

**Figure 4:**
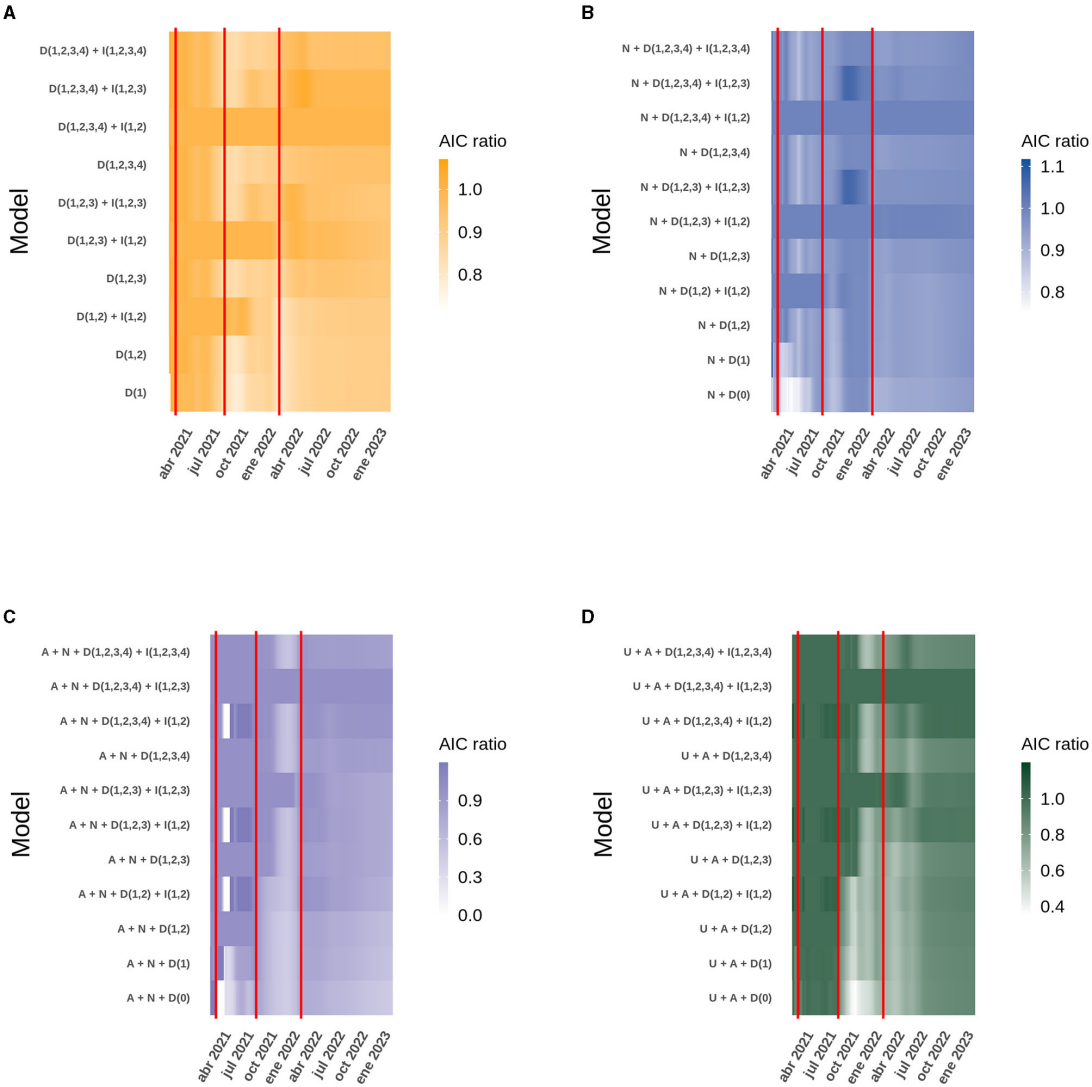
Heatmap of the AIC obtained daily from the best model compared to similar models for the data analyzed at the national level. The red vertical lines correspond to the dates on which the second, third and fourth doses of the vaccine were started. The letter code corresponds to: U (Daily ICU beds), A (Daily active cases), N (Daily new cases), D (Number of doses), I (Interaction between doses). **(A)** New cases. **(B)** Active cases. **(C)** ICU beds occupied by patients with COVID-19. **(D)** Deaths due to COVID-19.

The *R*^2^ obtained for each model, both at the national level and in the ICU bed models by age group, can be explained in the context of the start of the national vaccination campaign until February 8th, 2023. However, the analysis of each case does not explain how the model itself evolved over time and whether there were models with different variables that performed better in explaining the behavior of the data at certain points in time and, more importantly, how the interactions between the different doses explain the increase or decrease in performance of each model. For the national models, analyzing both new and active COVID-19 cases, all four doses are present in the model, but only the interaction between the first two doses gave the best model. The situation was similar for ICU bed occupancy and deaths, except that the interaction between the first three doses replaced the interaction with two doses. As time progressed, the models with more doses of vaccine differed from the others, but they were always accompanied by the interaction between the first and second dose for new and active cases, and the first three doses for ICU bed occupancy and deaths. It is also noteworthy that over time, models with few or no doses, and therefore fewer interactions, lose fitness, evaluated with the AIC, and ability to explain the dispersion of the data (*R*^2^) compared to themselves at the beginning of the national vaccination campaign. Finally, it can be seen that the model with the interaction between the four doses is not yet equal to the model with the interaction between the first three doses (Figure 2; Supplementary Figure 3). It can be added that in the models with more than three doses, the regression coefficients were similar, but the AIC was very pronounced, making it clear which is the best model, and this is more easily seen as the age range increases, suggesting the importance of the vaccination plan with a fourth dose in older adults (Figure 4; Supplementary Figure 4).

## Discussion

All the models presented in this report include the four vaccine doses as explanatory factors and support to their importance in reducing the severity of COVID-19 cases. All the selected models also include the factor of interaction between other variables. A statistical interaction is understood as a situation in which the effect of one causal variable on an outcome depends on the state of a second causal variable, i.e., when the effects of the two causes are not additive (23). In the context of the immunization of a population, we explain the presence of the four doses as the immediate and protective effect of vaccination through the production of antibodies and effector immune cells. However, there is also the factor of interaction between doses of vaccines, which we understand as the development of an immunological memory specific for viral antigens through immunization, and because this process establishes later in the development of an immune response. We have developed here models that consider four COVID19 vaccine doses given to the population and showed that only the interaction between the first two or three doses affected the data handling (Figures 2, 4). The interaction of the fourth dose is not present in the models of new cases, active cases and ICU beds occupied by patients between 3 and 59 years of age, due to the fact that people within this age range do not have great coverage with the fourth dose of vaccine preventing it from interacting at the population level with the other doses, but it is expected as the number of people vaccinated with this last dose is suspected, the interaction of the fourth dose appeared as an explanatory factor.

The model of new cases at national level shows that vaccination alone does not satisfactorily explain the decrease in cases, because the model does not include factors such as sanitary measures or variations in population mobility, and none of the models includes the different variants of the coronavirus circulating in each period.

With this work, we cannot confirm the mechanism by which heterologous vaccination works in the population, but based on studies, it has been seen that in the mouse model, the humoral and cellular immune response was poor when immunized with two doses of an inactivated virus vaccine, but the amount of neutralizing antibodies improved when a booster with an mRNA or adenoviral vector-based vaccine was applied (24), a heterologous adenoviral and mRNA vaccine schedule is better at developing a Th1 response in conjunction with cytotoxic T lymphocytes than a homologous vaccine schedule (25), and a recombinant BCG-based vaccine for the nucleoprotein has shown an increase in the number of CD4^+^ and CD8^+^ lymphocytes, and the parameters studied are related to a trained immunity profile (26). In Germany, a study showed that heterologous immunization with a first dose of the Oxford-AstraZeneca vaccine and a booster at 9–12 weeks with the Pfizer vaccine produced a higher level of neutralizing antibodies than homologous immunization with either vaccine (27), this is because the Pfizer vaccine induces a high production of antibodies, while the AstraZeneca vaccine induces a stronger cellular response, which when mixed together produces a much greater effect than vaccination with a single formulation (28). On the other hand, people who had two doses of CoronaVac^®^ and received a booster from Pfizer had higher levels of neutralizing antibodies specific to the beta, gamma and delta variants of SARS-CoV-2 than people who had a booster with CoronaVac^®^ again (29).

In Chile, a two-dose vaccination schedule with CoronaVac^®^ in children under 17 years of age was shown to be safe, with an increase in antibody titers and CD4^+^ lymphocyte activation 4 weeks after the second dose, although antibody titers against the Delta and Omicron (B.1.1.529) variants were lower than those against the D614G strain (30, 31). In a homologous schedule with a booster dose following two doses of CoronaVac^®^ vaccine in adults, an increase in neutralizing antibodies was observed 4 weeks after the booster dose, and an increase in anti-SARS-CoV-2 specific T cells was also observed, peaking 4 weeks after the booster dose (32). In addition, the immune response generated showed activity against Delta and Omicron (B.1.1.529) variants (33).

Our model is consistent with the study by Jara et al., which suggests that a homologous or heterologous booster dose for individuals with a complete primary vaccination schedule with CoronaVac^®^ provides a high level of protection against COVID-19, including severe disease and death. Heterologous boosters showed greater vaccine efficacy than homologous boosters for all outcomes (34).

A heterologous vaccination schedule has been shown to be more effective than a homologous vaccination, leading to the development of new vaccination schedules against this or other pathogens, and may also reduce the use of drugs for comorbidities (35).

The nature of these models is only explanatory but looking at how the models have behaved over time, we can predict that the models with the four doses and the interaction between the first, second and third dose will tend to be better than those already shown in this work, as long as the population completes its vaccination schedule with the four doses.

## Policy implications

This work highlights the importance of achieving full vaccination status and reinforces the notion that heterologous vaccination confers greater protection. The trends observed may also support the inclusion of seasonal vaccination program for vulnerable individuals. These data could guide other countries in their vaccination campaigns.

## Data availability statement

The original contributions presented in the study are included in the article/supplementary material, further inquiries can be directed to the corresponding author.

## Author contributions

HR: Data curation, Formal analysis, Methodology, Visualization, Writing—original draft, Writing—review and editing. CM: Visualization, Writing—original draft, Writing—review and editing. AK: Funding acquisition, Resources, Supervision, Writing—original draft, Writing—review and editing.
