# Supplementary material for: Statistical explanation of the protective effect of four COVID-19 vaccine doses in the general population
Source: Front Public Health. 2023 Sep 22;11:1253762. doi: 10.3389/fpubh.2023.1253762 (PMC10556658; doi:10.3389/fpubh.2023.1253762)
Supplement: Supplementary file 1 [file Data_Sheet_1.PDF]

Supplementary Figure

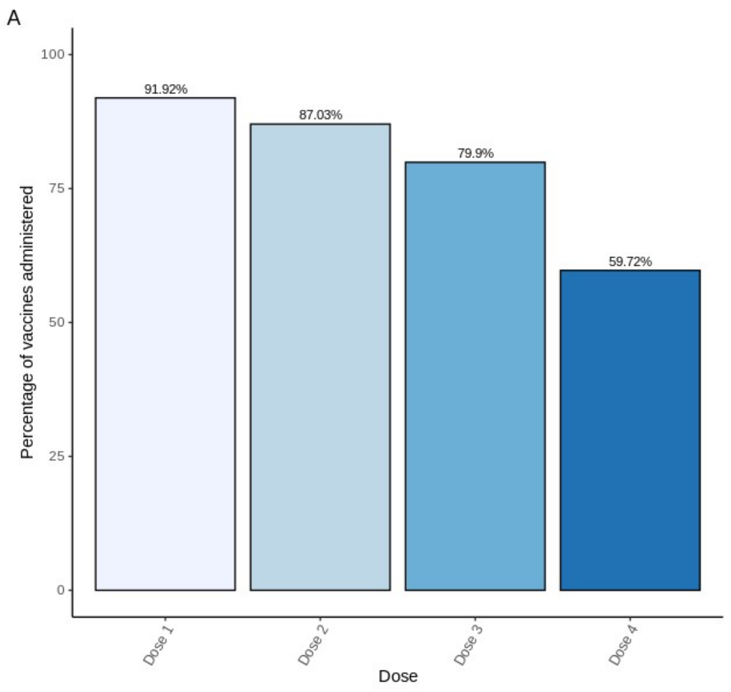

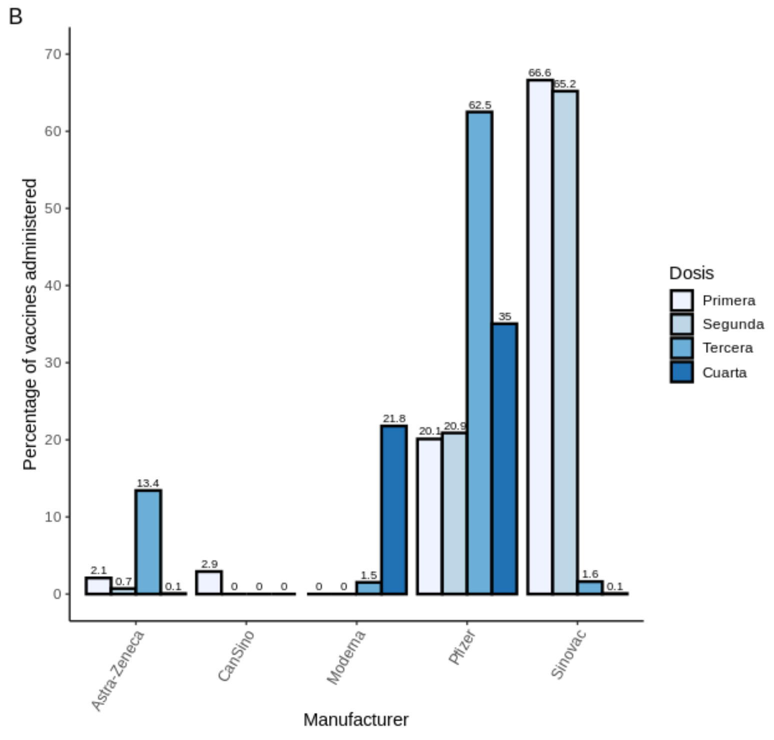

Supplementary Figure 1: Vaccines administered to the Chilean population from February 3, 2021 to February 3, 2023. A) Percentage of vaccines administered per dose. B) Percentage of vaccines administered per dose by manufacturer.

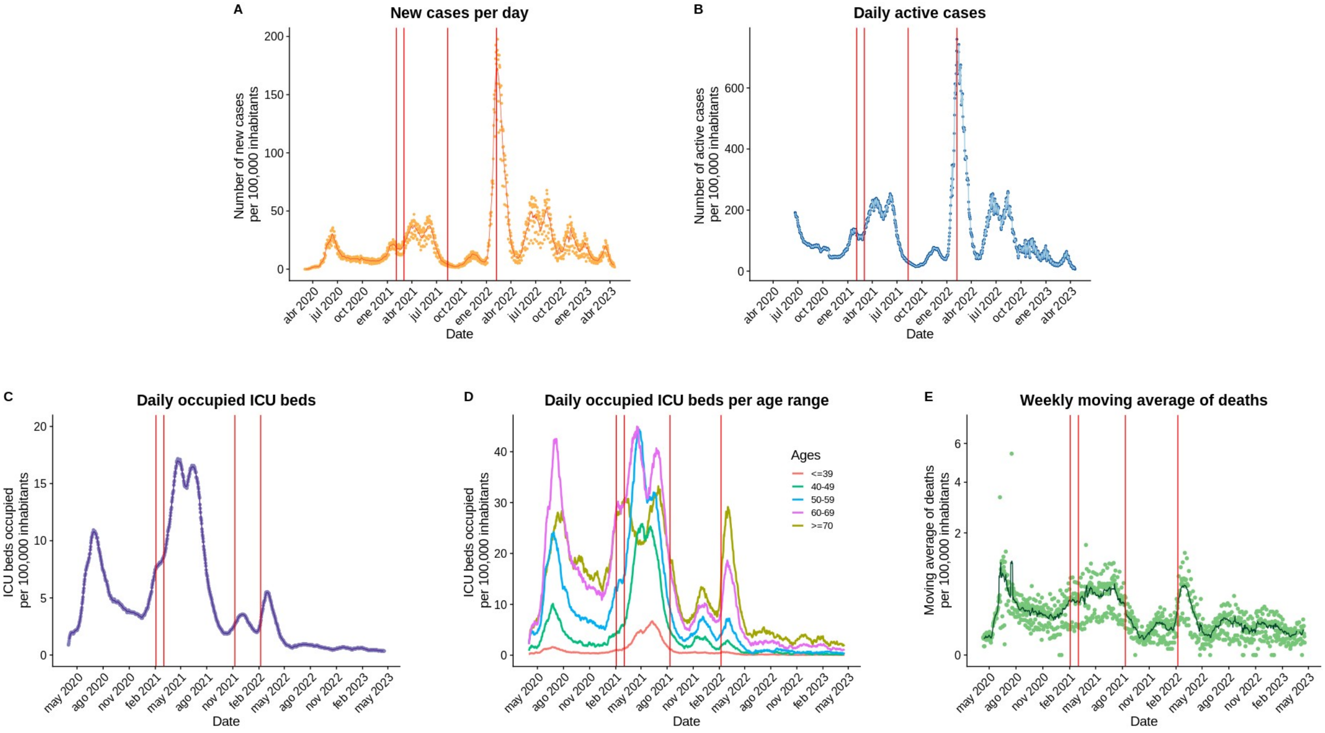

Supplementary Figure 2: National pandemic data provided by MINSAL. The dots correspond to the value of the daily data and the line through them corresponds to the weekly moving average of the data; the vertical red lines correspond to the starting date of the administration of each of the four doses of the vaccine. A) Daily new cases. B) Daily active cases, C) ICU beds occupied by patients with Covid-19. D) ICU beds occupied by age range. E) Confirmed deaths due to Covid-19.

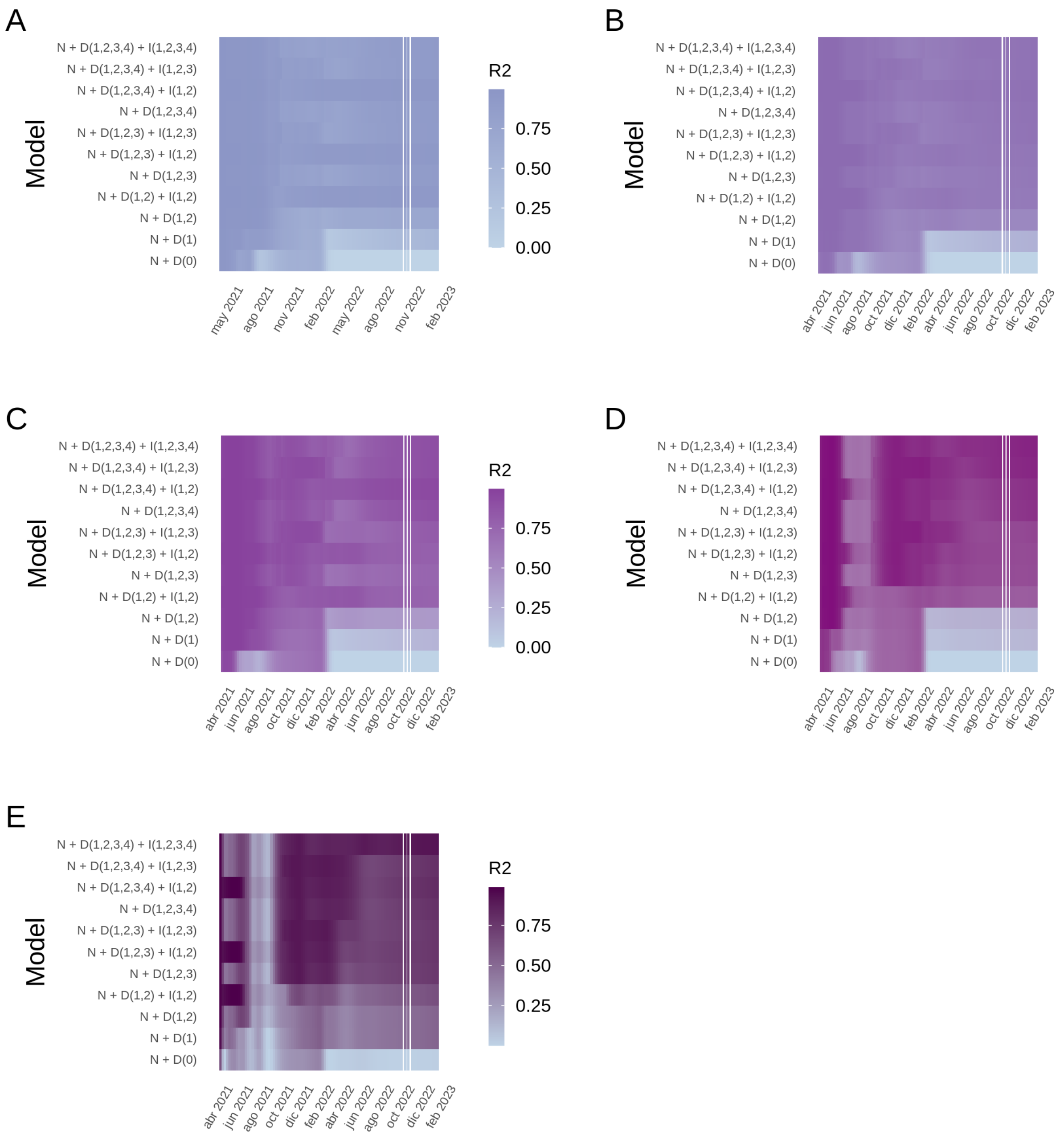

Supplementary Figure 3: Heatmap of the daily R^2 obtained from the best model compared to similar models for ICU beds by age range. The letter code is: U (Daily ICU beds), A (Daily active cases), N (Daily new cases), D (Number of doses), I (Interaction between doses): A) Persons younger than 39 years. B) Persons between 40 and 49 years of age. C) Persons between 50 and 59 years of age. D) Persons between 60 and 69 years of age. E) Persons over 70 years of age. The vertical lines shown are artifacts of the graph generation.

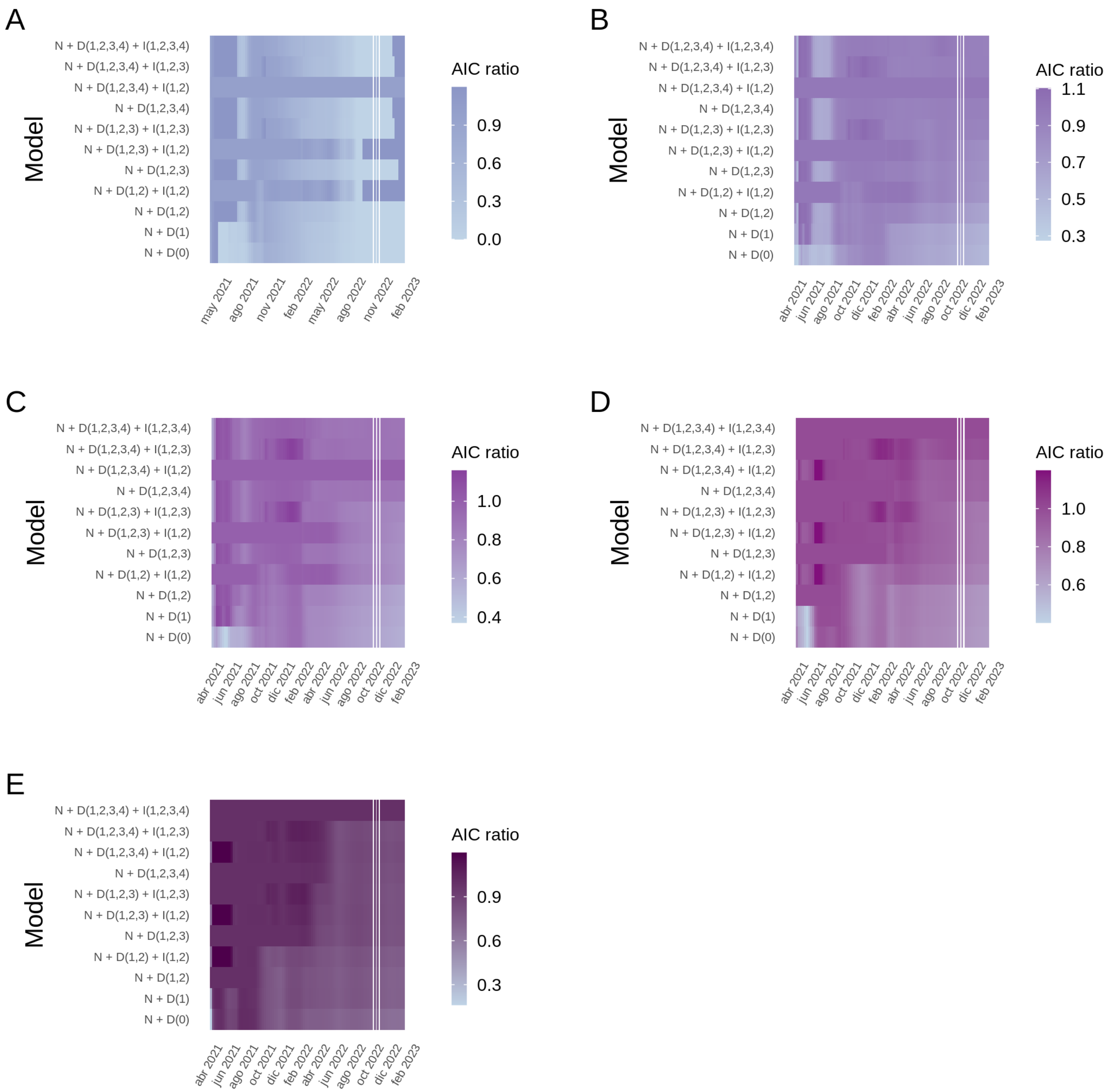

Supplementary Figure 4: Heatmap of the AIC obtained daily from the best model compared to similar models for ICU beds by age range. The letter code is: U (Daily ICU beds), A (Daily active cases), N (Daily new cases), D (Number of doses), I (Interaction between doses): A) Persons younger than 39 years. B) Persons between 40 and 49 years of age. C) Persons between 50 and 59 years of age. D) Persons between 60 and 69 years of age. E) Persons over 70 years of age. The vertical lines shown are artifacts of the graph generation.
